# Factors associated with the health status of internally displaced persons in northern Uganda

**DOI:** 10.1136/jech.2008.076356

**Published:** 2008-11-21

**Authors:** B Roberts, K Felix Ocaka, J Browne, T Oyok, E Sondorp

**Affiliations:** 1 Conflict and Health Programme, Health Policy Unit, Department of Public Health and Policy, London School of Hygiene and Tropical Medicine, UK; 2 Faculty of Medicine, Gulu University, Gulu, Uganda; 3 Health Services Research Unit, Department of Public Health and Policy, London School of Hygiene and Tropical Medicine, UK

## Abstract

**Background:**

Globally, there are over 24 million internally displaced persons (IDPs) who have fled their homes due to violence and insecurity but who remain within their own country. There have been up to 2 million IDPs in northern Uganda alone. The objective of this study was to investigate factors associated with mental and physical health status of IDPs in northern Uganda.

**Methods:**

A cross-sectional survey was conducted in November 2006 in IDP camps in the Gulu and Amuru districts of northern Uganda. The study outcome of physical and mental health was measured using the SF-8 instrument, which produces physical (PCS) and mental (MCS) component summary measures. Independent demographic, socio-economic, and trauma exposure (using the Harvard Trauma Questionnaire) variables were also measured. Multivariate regression linear regression analysis was conducted to investigate associations of the independent variables on the PCS and MCS outcomes.

**Results:**

1206 interviews were completed. The respective mean PCS and MCS scores were 42.2 (95% CI 41.32 to 43.10) and 39.3 (95% CI 38.42 to 40.13), well below the instrument norm of 50, indicating poor health. Variables with negative associations with physical or mental health included gender, age, marital status, income, distance of camp from home areas, food security, soap availability, and sense of safety in the camp. A number of individual trauma variables and the frequency of trauma exposure also had negative associations with physical and mental health.

**Conclusions:**

This study provides evidence on the impact on health of deprivation of basic goods and services, traumatic events, and fear and uncertainty amongst displaced and crisis affected populations.

There are over 45 million persons worldwide who have been forcibly displaced from their home areas due to conflict and insecurity.^1^ ^2^ Over 24 million of these are internally displaced persons (IDPs) who have remained within the boundaries of their own country.^1^ Research on the health of IDPs has focused upon specific physical and mental health conditions. Little is known about the determinants of overall health status.

The objective of this study was to investigate factors associated with health status of IDPs in northern Uganda. The study adopted a broad comprehension of health including physical, mental, emotional and social characteristics. Northern Uganda was selected for the study because up to 2 million IDPs have been displaced as a result of the 20-year conflict waged between a rebel group, the Lord’s Resistance Army, and the central government. The IDPs are largely based in the most conflict-affected districts of Gulu, Amuru, Kitgum and Pader, which are mainly populated by the Acholi people. Approximately 85% of the IDPs in northern Uganda live in government-organised camps established to protect civilians and aid the government’s counter-insurgency campaign.^3^ The camps are characterised by chronic over-crowding, insecurity, social problems and illness.^4^^–^6

## METHODS

The study took place in November 2006 in the Gulu and Amuru districts of northern Uganda. The two districts contain an estimated 650 000 IDPs, which is approximately 40% of all IDPs in Uganda. Up to 80% of the districts’ population live in camps that range in size from 1100 to almost 60 000.^7^ ^8^ A cross-sectional survey design was used. The sampling population was adult (⩾18 years old) male and female IDPs. IDPs were defined as people living in officially recognised camps.

### Study questionnaire

Within the study questionnaire general physical and mental health were measured using the 4-week recall version of the SF-8, which is a psychometric instrument developed by QualityMetric.^9^ The instrument seeks to measure general physical and mental health status and is not specific to age, disease or health condition. The SF-8 has single item scales and consists of eight items that measure the eight health domains used in a number of longer, related instruments such as the SF-12 and SF-36. A summarised version of the eight domains and their respective items is given in box 1.

**Box 1 Summary of SF-8 domains and questions**

1. General health: How would you rate your health?
2. Physical functioning: How did physical health problems limit usual physical activities?
3. Role limitation (physical): How much did physical health problems limit your daily work?
4. Bodily pain: How much bodily pain have you had?
5. Vitality: How much energy did you have?
6. Social functioning: How much did physical or emotional problems limit your usual social activities?
7. Mental health: How much have you been bothered by emotional problems?
8. Role limitation (emotional): How much did emotional problems limit your daily activities?

Physical (PCS) and mental (MCS) component summary measures are produced from the individual items. These are calculated by weighting each SF-8 scale using norm-based scoring methods and weights derived from the initial validation work for this instrument.^9^ Higher summary PCS and MCS scores are indicative of better health. Scores above and below 50 are considered above and below the average in the general US population.^9^

The SF-8 has been translated in over 30 different languages, and used in a number of countries.^9^^–^12 Related, longer instruments such as the SF-36 have been successfully used with conflict-affected and low-income populations, but mainly to measure specific dimensions such as social functioning.^13^^–^18 The SF-8 showed generally strong reliability and validity for use with IDPs in northern Uganda.^19^

Items on various possible independent variables affecting health status were included in the study questionnaire. The selection of independent variables was informed by a systematic literature review and consultations with humanitarian and academic staff from Gulu, Kampala, Geneva and London. The selected independent variables were: age, religion, ethnicity, education level, period of displacement, number of camps lived, distance of camp from home area, number of persons per household, latrine use, water source, hygiene/use of soap, food security, income source and amount, health knowledge, utilisation of health services, sense of safety in camps and alcohol intake (using the AUDIT instrument^20^). Sixteen items were included on lifetime exposure to traumatic events using a slightly modified version of the original Harvard Trauma Questionnaire (HTQ). The HTQ was developed and has been used in conflict-affected settings.^14^ ^21^^–^24 The study questionnaire also contained items measuring self-reported physical health conditions, and items on post-traumatic stress disorder and depression using the HTQ and the Hopkins Symptoms Checklist-25^21^ ^25^ (results reported elsewhere^26^).

The questionnaire was translated and delivered in Luo, the main language of the Gulu and Amuru districts. The translation followed recommended guidelines, and involved forward and back translation, pre-testing and detailed review by the study team.^21^ ^27^ ^28^ Pre-testing to pilot the questions and ensure the accuracy of the translation was conducted with a sample of IDPs in a camp not included in the main survey.

### Sampling procedure

A minimum sample size of 1080 was required to detect associations of independent variables on the SF-8 outcome. This required adequate power (80%) to detect conceptually important differences (0.8 standard deviation) in the health outcomes within a multivariate analysis with a significance level of 5% with the size of the “rarest” sub-group of respondents at 5%.^29^ To account for the clustered design, a design effect of 2.0 was included, which doubled the required sample size.^30^ The expected proportion of unusable questionnaires was set at 10%.

An adapted multi-stage cluster sampling method was used.^30^ The first stage randomly selected clusters from which the camps would be chosen. The sampling frame was a list of the total population of IDPs living in all the 65 officially recognised IDP camps in the Gulu and Amuru districts.^8^ Thirty-two clusters were chosen rather than the more common use of 30 clusters to reduce the design effect.^31^ The 32 clusters were selected using the probability proportional to size technique to self-weight the clusters.^30^ The 32 clusters were allocated to 28 IDP camps using this process. The total population living in the 28 selected camps was 452 702.

Due to the large population size of the camps, a second stage was used to randomly select administrative zones within the sampled IDP camps to serve as clusters. These were estimated to be of similar population sizes based upon discussions with camp and zone leaders, and so self-weighting was maintained. The third stage consisted of randomly choosing individuals from the selected clusters. As the clusters were already self-weighted, the same number of individuals were chosen from within each of the selected clusters. The Expanded Programme on Immunisation (EPI) method was used to randomly select households for this stage and one individual was then randomly selected from the eligible individuals within the household.^31^^–^33 Two study staff conducted stage 2 and 3 of the sampling process through a “pre-visit” one or two days prior to the actual day of data collection. In stage 3, if the randomly selected person was not present, another adult member of the household or a neighbour was asked to inform the selected person to attend the data collection. It was advised that replacements would not be accepted and precautions were taken to reduce risk of replacements.

A team of 15 data collectors were recruited for the survey (eight men and seven women) who were all from the Acholi region of northern Uganda and spoke fluent Luo and English. Six days training was provided for the study. The data collection took place between 6 and 27 November 2006. Each interview took approximately 35–45 minutes.

### Ethics

Ethical approval for the study was provided by the Ugandan National Council for Science and Technology, Gulu University, and the London School of Hygiene and Tropical Medicine. A consent form was used to ensure informed consent and clarify that no direct benefit could be expected from participating in the study. All data collected were confidential and anonymous. As some of the questions were on traumatic experiences, referral information for support on mental health was provided. One of the study team was a psychiatrist and one of the team leaders was a double-trained Clinical Psychiatric Officer/Mental Health Nurse who could offer advice if required. Supervision and quality control were provided by the three members of the study team and two team leaders.

### Statistical analysis

Two data entry clerks were used to enter the data into SPSS, version 14.0 (SPSS Inc, Chicago, USA). All analysis was performed using STATA version 9.2 (Stata Corporation, College Park, Texas, USA) and adjusted for the clustered design. A univariate linear regression analysis was firstly conducted to explore associations between all independent variables on PCS and MCS. The independent variables that showed statistically significant (p<0.05) associations were then included for multivariate linear regression analysis, and adjusted to address the influence of the other significant variables. This followed a stepwise approach with forward selection and backward regression.^34^ The statistically significant variables (p<0.05) remaining in the model were included in the final results. Positive coefficients indicate a positive association with higher PCS or MCS scores (i.e. better physical or mental health). Negative coefficients indicate a negative association with PCS or MCS scores (i.e. worse physical or mental health). Interaction tests were conducted to explore if the effect of one independent variable on PCS or MCS significantly varied (p<0.05) according to the level of the effects of another independent variable within the overall regression model.

## RESULTS

The total number of completed individual interviews was 1206. The response rate was 94%. There were 44 absent individuals, 22 non-consenting individuals, and 8 incomplete interviews.

The sample characteristics are summarised in table 1. A greater proportion of women (60%) than men were in the sample. The mean age of respondents was 35 years. Over three-quarters of respondents were married/co-habiting (77%). Over two-thirds (70%) of respondents had been displaced for 5 or more years. Nearly two-thirds (61%) of respondents earned less than 50 000 Ugandan shillings (approximately 28 US dollars) in the month preceding the study.

**Table 1:** Summary sample characteristics of Ugandan IDP respondents (n = 1206)

Variable	Number (%)
Demographic and social	
Number of women	724 (60.0)
Mean age	35 years
Acholi ethnic group	1096 (90.9)
Attended school (primary or higher)	828 (68.7)
Marital status:	
married/co-habiting	924 (76.6)
separated*	210 (17.4)
single	72 (6.0)
Income <UGS 50 000 in previous month	958 (79.4)
Period since forced to camp(s):	
<2 years	88 (7.3)
2–5 years	275 (22.8)
5–10 years	479 (39.7)
>10 years	364 (30.2)
Number of camps lived in:	
one camp	709 (58.8)
two camps	384 (31.8)
three or more camps	113 (9.4)
Mean distance of camp from home area	6.2 miles
>5 persons living in household	732 (60.7)
Food in household for <5 days	201 (16.67)
Water use includes unprotected source†	146 (12.1)
Currently have soap in household	289 (24.0)
>20 people sharing a latrine	256 (21.2)

Abbreviations: Ush, Ugandan shillings (1USD  =  1800 Ush at time of survey).

*Separated includes divorced/separated, widowed, forced separation.

†Unprotected water source includes well, stream, river, lake, pond.

Table 2 provides data on the exposure to traumatic events experienced by respondents. Over half (58%) of respondents had experienced 8 or more of the 16 trauma events covered in the questionnaire. Over half of all the traumatic events listed in table 2 occurred in the time-period when the participants were living in a camp (the detailed trauma results are reported elsewhere^26^). Ninety-three per cent of respondents did not feel safe in the camp. The main reasons cited for this perceived poor safety were lack of food (62%), fear of disease (61%), and insecurity and fear of armed forces (54%).

**Table 2:** Exposure to traumatic events of Ugandan IDP respondents (n = 1206)

Trauma events experienced	
Lack of food or water	1085 (89.9)
Lack of housing or shelter	931 (77.2)
Unnatural death of family/friend	913 (75.7)
Murder of family member/friend	902 (74.8)
Being close to, but escaping, death	841 (69.7)
Ill health without medical care	784 (65.0)
Witnessing murder of stranger(s)	775 (64.3)
Tortured or beaten	678 (56.2)
Forced separation from family	546 (45.3)
Being abducted or kidnapped	521 (43.2)
Made to accept ideas against will (brainwashing)	482 (39.9)
Serious injury	473 (39.2)
Forced isolation from other people	450 (37.3)
Being in a war fighting situation	329 (27.3)
Imprisonment against your will	296 (24.5)
Rape or sexual abuse	171 (14.1)

The mean SF-8 scores are provided in table 3. Women had lower PCS and MCS scores than men. These MCS and PCS scores are substantially lower than the norm for the US general population of 50. 9

**Table 3:** Mean SF-8 scores of Ugandan IDP respondents (n = 1206)

Mean SF-8 score (95% CI)	
All sample PCS	42.2 (CI 41.54 to 42.89)
Men PCS	43.8 (CI 42.74 to 44.85)
Women PCS	41.2 (CI 40.29 to 42.03)
All sample MCS	39.3 (CI 38.55 to 40.00)
Men MCS	41.0 (CI 39.87 to 42.20)
Women MCS	38.1 (CI 37.18 to 39.02)

Abbreviations: CI, confidence interval; PCS, physical component summary; MCS, mental component summary.

The three main physical health conditions reported were fever/malaria (48%), respiratory problems (45%) and watery or bloody diarrhoea (22%). Over half (54%) of respondents met symptom criteria for post-traumatic stress disorder, and over two-thirds (67%) of respondents met symptom criteria for depression (the detailed results of post-traumatic stress disorder and depression are provided elsewhere). The main sources of support for people experiencing emotional distress were friends (56%), family (50%), religious leaders (19%) and health staff (10%).

### Multivariate analysis

The independent variables with statistically significant (p<0.05) associations with PCS and MCS in multivariate linear regression analyses are presented in table 4. Independent variables with a negative association with PCS were female gender, increasing age, being separated (divorced/separated, widowed, forced separation), increasing distance of camp from home area, absence of soap in the household, and exposure to traumatic events (serious injury, ill health without medical care, narrowly escaping death).

**Table 4:** Multivariate analysis for social variables and health variables

Variables	n	PCS		MCS	
		Coef. (95% CI)	p Value	Coef. (95% CI)	p Value
Socio-demographic variables:					
sex (women)	724	−2.94 (−4.62 to −1.26)	<0.01	−2.11 (−3.84 to −0.37)	0.02
age	1206	−0.11 (−0.17 to −0.06)	<0.01	−	−
marital status (separated)‡	210	−2.04 (−3.96 to −0.12)	0.04	−2.33 (−4.38 to −0.28)	0.03
no soap	289	−2.57 (−4.01 to −1.13)	<0.01	−	−
camp >5 miles from home area	377	−0.09 (−0.11 to −0.06)	<0.01	−	−
monthly income <50 000 Ush	958	−	−	−2.01 (−3.77 to −0.25)	0.03
food in household for <5 days	201	−	−	−2.84 (−4.99 to −0.69)	0.01
do not feel safe in camp	1122	−	−	−3.69 (−6.60 to −0.78)	0.02
use family for emotional support	677	−	−	2.26 (0.64 to 3.89)	0.01
Trauma events variables:					
ill health without medical care	784	−2.74 (−3.79 to −1.70)	<0.01	−3.69 (−5.31 to −2.07)	<0.01
serious injury	473	−3.10 (−4.83 to −1.37)	<0.01	−	−
made to accept ideas against will	482	−	−	−3.12 (−4.77 to −1.46)	<0.01
murder of family member/friend	902	−	−	−2.91 (−4.73 to −1.08)	<0.01
lacked food/water	1085	−	−	−2.68 (−5.16 to −0.20)	0.04
narrowly escaping death	841	−2.25 (−3.47 to −1.04)	<0.01	−	−
rape or sexual abuse	171	−	−	−1.93 (−3.81 to −0.05)	0.05
Cumulative trauma events:†					
0–3 trauma events	103	Ref	−	Ref	−
4–7 trauma events	407	−1.32 (−3.80 to 1.17)	0.30*	−5.04 (−7.68 to −2.39)	<0.01
8–11 trauma events	422	−3.22 (−5.71 to −0.74)	0.01	−8.08 (−10.73 to −5.43)	<0.01
12–16 trauma events	274	−5.11 (−7.77 to −2.45)	<0.01	−12.07 (−14.90 to −9.24)	<0.01

Abbreviations: PCS, physical component summary; MCS, mental component summary; Coef., coefficient; CI, confidence interval; Ush, Ugandan shilling (1USD  = 1800 Ush at time of survey); ref, reference group.

−Not included as not statistically significant (p>0.05).

*Not statistically significant (p*>*0.05).

Statistically significant (p<0.05) independent variables from univariate analysis included in multivariate analysis. Only statistically significant variables from multivariate analysis presented (except *).

†Frequency of trauma events analysed separately from individual trauma exposure variables in multivariate analysis.

‡Separated included includes divorced/separated, widowed, forced separation.

Independent variables with significant negative associations with MCS were female gender, being separated, having food for less than 1 week, having a monthly income of less than 50 000 Ugandan shillings, inability to discuss emotional problems with family members and exposure to traumatic events (ill health without medical care, being forced to accept ideas/brainwashing, murder of family or friend, lack of food or water, and experiencing rape or sexual abuse).

In separate analyses, higher frequency of trauma exposure showed a strong significant negative association with PCS and MCS.

No significant interaction effects between independent variables were found.

## DISCUSSION

This study presents evidence on the overall physical and mental health status of persons displaced by conflict in northern Uganda, and the significant association of demographic, socio-economic, and trauma event variables upon overall physical and mental health. It makes an important contribution to work investigating social determinants of health in conflict- and crisis-affected countries.^35^

Gender and marital status had a strong negative association with physical and mental health. Women have lower scores than men in other studies using the SF-8 in stable settings, but the differences are not as wide as in this study.^9^ The influence of gender and marital status on specific mental health outcomes such as post-traumatic stress disorder and depression amongst conflict-affected populations has been recorded in other studies, and may be partly attributable to the psychological consequences of rape, the violent loss of partner and children, and of becoming a single parent or widow.^14^ ^15^ ^36^^–^38

The negative association of distance from home areas with physical health may be explained by reduced access to land to farm food for personal consumption and commercial use, which could directly and indirectly affect physical health. Absence of soap from a household also had a negative association with physical health, and this could contribute to diarrhoeal disease and worsening physical health. Soap may also act as a proxy for other factors not captured in the analysis. Variables of income (<50 000 Ush) and food security (food<5 days) showed a negative association with mental health. This could be explained by the uncertainty and anxiety caused by limited income and food insecurity and has also been noted in studies on specific mental health conditions.^13^

The study provides evidence on the effects of traumatic events on both physical health and mental health. Traumatic events with significant associations on worse physical health included being ill without access to medical care, narrowly escaping death and being seriously injured. Traumatic events associated with worse mental health include the murder of family/friend, narrowly escaping death, being forced to accept ideas (brainwashing), rape and sexual abuse. The negative association of these variables correspond with studies on specific mental health outcomes.^14^ ^37^ The negative association of trauma variables such as illness without access to medical care and deprivation of food and water with mental health highlight the strong effect that the absence basic goods and services can have upon mental health. A number of sources of emotional support were reported by respondents, but the only one that showed a significant association with better mental health was support by family members.

There is a strong association between frequency of exposure to trauma events with poor physical health and mental health. The dose–response relationship between trauma exposure and poor mental health is consistent with studies on PTSD and depression amongst displaced population.^14^ ^15^ ^37^ ^39^ ^40^ Many of the trauma variables reported took place in the camps, and the variable on perceived lack of safety in the camps had a strong negative association with mental health. The study results provide evidence of the importance of ensuring adequate protection in camp situations to reduce exposure to traumatic events and poor mental health outcomes.

No significant interaction effects between independent variables were found. The lack of moderating influence by other independent variables suggests a strong, pervasive association between the significant variables (table 4) and PCS and MCS.

### Limitations

This study has a number of limitations. The cross-sectional design means observed relationships between variables were descriptive and not causal. The study was unable to consistently match the gender of interviewer and respondents. As a result, there may have been underreporting of certain sensitive traumatic events. Information on non-responders was not collected. The validity of measuring generic and mental health outcomes in different cultural settings has been questioned.^41^ ^42^ However, the SF-8 has been translated in over 30 different languages, and used in a number of countries.^9^^–^12 It also showed generally strong levels of reliability and validity in this study.^19^ The related SF-36 has also been successfully used in Sub-Saharan Africa and also with conflict-affected populations.^13^–^18^ ^43^^–^46

## CONCLUSIONS

This is one of the first studies investigating the factors that affect the overall physical and mental health of IDPs. It provides data on the impact of deprivation of basic goods and services, traumatic events, and fear and uncertainty on overall physical and mental health amongst displaced and crisis-affected populations, and evidence of the need for greater assistance and protection for IDPs in northern Uganda.

**What is already known on this subject**

Research on the health of internally displaced persons has focused upon specific health conditions. Very little is known on the effect of violent conflict and internal displacement on overall health.

**What this study adds**

This is one of the first studies to quantify the factors that influence the overall physical and mental health of internally displaced persons. This study provides evidence on the impact of displacement and deprivation of basic goods and services on both physical and mental health. The study also found a strong association of the frequency and type of exposure to traumatic events on both poor physical and mental health. The study calls for greater assistance and protection for internally displaced persons to improve overall health.
